# Pavement policies: unraveling the Norwegian ban on skateboarding

**DOI:** 10.3389/fspor.2024.1488825

**Published:** 2025-01-06

**Authors:** Tommy Langseth, Nils Asle Bergsgard

**Affiliations:** Department of Sports, Physical Education and Outdoor Studies, Faculty of Humanities, Sports and Educational Sciences, University College of South-Eastern Norway, Bø, Norway

**Keywords:** skateboard, policy regulations and processes, prohibitions, risk management, subculture

## Abstract

This paper investigates the historical prohibition of skateboarding in Norway from 1977 to 1989, a unique instance of such a comprehensive ban globally. The study aims to understand the circumstances leading to this ban and the rationale behind it. Two primary explanations emerged around the ban: one from a bureaucratic perspective citing risk management, and the other from skateboarders seeing it as a regulation of their counterculture. We argue that neither narrative alone is sufficient, proposing instead that other mechanisms were at play. Firstly, the ban was the inaugural case under the newly enacted Product Control Act, which was initially designed to address environmental issues. The State Pollution Control Authority found itself ill-prepared to handle the new responsibilities inherent in product control, resulting in diffuse responsibilities across several agencies. Secondly, the ambiguous categorization of skateboards as toys rather than sports equipment influenced the decision to enact the ban. The timing of the skateboard phenomenon coincided with the passing of the Product Control Act, suggesting a case of a solution seeking a problem. In conclusion, we posit that the skateboard ban resulted from a complex interplay of factors, including novel legislation, ambiguous responsibilities, cultural categorizations, and coincidental timing, rather than being solely a response to risk management or counterculture curtailment.

## Introduction

The phrase “Skateboarding is not a crime” is a common sight on bumper stickers, t-shirts, and skateboard decks, across the globe. While this assertion can be regarded as a primary element in the formation of an internal identity within skateboarding subcultures, the focus of this paper is on the historical reality of skateboarding being classified as a criminal act. From 1977 to 1989, the use of skateboards was prohibited in Norway. It was unlawful to possess, utilize, sell, or distribute skateboards. Norway was the sole nation to implement a comprehensive prohibition on skateboarding.

The objective of this paper is to examine the circumstances that led to the implementation of this ban and to gain insight into the rationale behind it. Two main narratives have emerged regarding the reasons for the prohibition of skateboarding. From a bureaucratic perspective, the ban was justified by citing the purported risks associated with skateboarding. These risks were perceived to affect not only the skateboarders themselves but also pedestrians and the urban environment. For those who engaged in skateboarding, the ban was not perceived as an effort to mitigate risk; rather, it was regarded as a means of regulating an irreverent and oppositional culture.

We put forth the proposition that neither of these narratives is sufficient in and of itself. Instead, we propose that, building on neo-institutional theory, other mechanisms were at play when the ban was first introduced. Firstly, we argue that the prohibition of skateboarding in Norway, can be related to risk management, and more so than to an attempt to curtail the counterculture. Secondly, that the ultimate result of this, namely a total ban, can be seen as a consequence of bureaucratic categorization and institutional logics within the context of Norway's regulatory environment. The paper thus represents a theoretical investigation of a specific policy process, rather than a historical study of skateboarding in Norway. It offers insights into sociological studies of the sport field, particularly the question of inclusion and exclusion, as well as studies of decision-making in policy more generally. In order to provide a comprehensive analysis, it is essential to consider both the international context, including the various attempts to regulate skateboarding in different countries, and the national context, specifically the Norwegian political climate at the time.

### The Norwegian skateboard ban

The regulation of skateboarding is a phenomenon that has persisted for nearly as long as the sport itself. As early as the 1960s, the California Medical Association voiced concerns about skateboarding, leading to legislative restrictions imposed by city councils (1). The regulations were legitimized by invoking the perceived dangers of skateboarding, both to the skaters themselves and to pedestrians and other individuals sharing the same public spaces (1). Moreover, skateboarding was perceived as a potential hazard to the urban environment. Consequently, skateboarding has been subjected to a variety of regulatory frameworks in numerous countries. The methods of regulating skateboarding have varied considerably. These include the enactment of bylaws that prohibit skateboarding in specific urban areas and the introduction of devices such as “skatestoppers”, which effectively render skateboarding impossible (2–4). The literature on how skateboarders are excluded from urban areas by laws and regulations is often understood in the light of counter-cultural (implicit) critiques of neoliberalism and capitalism an efforts made to curtail transgressive behavior (1, 5–7). However, as Carr (8) points out “(…) my research suggests that the aggressive singling out of skateboarders for regulatory exclusion from the urban core is as much a product of the clash between logics of private property and the transgressive, potentially destructive practices of street skater”. More than singling out skaters as “problem youth”, the regulatory framework around skating in many urban areas around the world is based on different kinds of property laws. The Norwegian national ban on skateboarding, on the other hand, was, as we shall see, handled under completely different law systems, namely environmental and product control laws that has little or nothing to do with either property laws, regulation of urban space or exclusion of unwanted irreverent groups of youth.

As posited by Peter Wagner (9), risk management constituted a pivotal element in the advent of the welfare state. As social policy developed in Europe throughout the nineteenth century, the concept of risk management shifted from an individual to a collective responsibility. Wagner posits that the welfare state can be conceptualized as a political technology for risk management. At the core of Wagner's argument lies the assertion that the pursuit of individual liberty and the imposition of discipline have been inextricably intertwined throughout the history of modernity. In certain historical periods, the concepts of liberty and discipline have held sway in opposing directions.

In the period following the Second World War, which has been characterized as the “social democratic era” or “social democratic order” in Norway (10), “discipline” has been a dominant force, particularly during the 1950s and 1960s. This era was characterized by a strong state, which emphasized equality over liberty and thus regulation over freedom of choice. Towards the end of the 1970s, the process of dismantling the social democratic order commenced. The previous governing regime, which sought to establish a counter-power to the power of the market, was replaced by a new governing regime that sought solutions through the market, according to Slagstad (11). The prohibition of skateboarding can thus be regarded as an illustration of what has been termed the “patronage state”, but it may also be seen as indicative of the decline of an era. The argument against unwarranted governmental interference from a “patronage state” foreshadowed the neoliberal turn of the 1980s, exemplified by the administrations of Reagan in the United States and Thatcher in the United Kingdom. In Norway, the Conservative Party (Høyre) assumed control of the government in 1981, which resulted in an acceleration of the deregulation and liberalization of various sectors of Norwegian society. Nevertheless (12), work, Three Worlds of Welfare Capitalism, continues to categorize Norway, along with its Nordic neighbors, as a “social democratic welfare regime”.

The regulatory framework governing risk sports in Norway is characterized by a certain ambivalence. For an extended period, professional boxing was prohibited in Norway. However, this prohibition has since been revoked. Nevertheless, professional MMA remains prohibited, as the rationale for this prohibition is based on the same argument regarding the objective of the game, namely, to knock out one's opponent, and the inherent risk of head injuries that this entails. Conversely, certification is not a prerequisite for participation in activities such as rock climbing or white-water paddling. Some activities are subject to a certain degree of regulation. Nevertheless, other activities that may be considered dangerous, such as base jumping, are permitted in most locations within Norway. Consequently, the Norwegian approach to the regulation of risk in sport is relatively liberal, particularly when the risk element is connected to the athlete himself and not his opponent. In light of this, we posit that an additional explanation for the prohibition of skateboarding is necessary, beyond the factors of risk management or moral panic. This additional explanation must take into account the political process that preceded the ban.

### Theoretical Lens

As previously stated, Wagner asserts that modernity is characterized by a conflict between freedom and discipline. He further suggests that the welfare state can be conceptualized as a political technology for risk management, with a greater emphasis on the discipline side. Nevertheless, in a more rationalized and routinized society, there should still be scope for more transgressive behavior within institutional frameworks. This can be seen as a “quest for excitement in an unexciting society”, as Elias and Dunning (13) put it. It is not the case that the welfare state does not provide opportunities for those seeking pleasure. However, the reduction of risk in everyday life still creates a psychological need for more emotional expression (14). Accordingly, in Elias's (15) perspective, sport occupies a pivotal role in the process of civilization. This is evident in two key ways: firstly, by transforming more extreme and violent folk games into formalized sports with established rules and regulations; and secondly, by offering a platform for emotional and physical expression within a rationalized lifeworld. This encompasses actions that, in the absence of the context of sport, would be classified as violent and thus carry the risk of injury and pain. This paper will demonstrate that the ambiguity surrounding the categorization of the skateboard—is it a toy or sports equipment? —is an important feature that underlines the ban. Skateboarding was regarded as play, and thus the potential for injury was seen as problematic. In contrast, risk and injury in the context of sport were viewed as inherent to the nature of the activity. Furthermore, sport is viewed as a serious endeavor in Norway.

Secondly, in order to gain insight into the rationale behind the skateboard ban, it is essential to examine the decision-making process that led to this outcome. Bureaucratic-administrative decisions are typically perceived as rational and the decision-making process as comprising a series of logical steps. If the grounds for the decision are known, the result should be apparent. Many organizational theorists, often referred to as “neo-institutionalists”, have challenged this “closed system logic”, arguing that organizations and decision-making processes should be seen as open systems. The analysis of decisions is influenced by a number of factors, including institutional norms, the “logic of appropriateness”, and the cognitive constitution of the situation (16).

One of the approaches that challenges the assumption of a rational, logical decision-making process is put forth by March and Olsen. They argue that “we often have underestimated the extent to which choice situations in organizations involve problematic goals, unclear technologies, and fluid participation” (17). To comprehend the nature of choice and decision-making in this context, March and Olsen posit that they are “garbage can processes” [see also (18), where this model was initially presented]. The decision-making process can be conceptualized as a series of four interdependent elements: (1) the choices made by the system, (2) the problems that these choices aim to address, (3) the solutions that can be applied to resolve the problem, and (4) the participants who bring diverse perspectives about the problem and the solution. Timing is a crucial factor in this context; it could be argued that the temporal order supersedes the causal order. Such decisions may be relatively arbitrary in nature.

Subsequently, this model has been further developed and refined under the umbrella term Multiple Streams Framework (MSF) of policy agenda setting and decision making (19–21). MSF elucidates some fundamental tenets of policy-making;

• Ambiguity: The lack of clarity regarding the nature of the problem.
• Time constraints: The necessity to make a decision within a specified timeframe.
• Incomplete preferences: The act of selecting an option that may not be optimal, but is nevertheless appropriate.
• Unclear technology: The lack of clarity regarding the boundaries of authority and responsibility.
• Fluid participation: The involvement of multiple actors from diverse organizations.

In essence, the MSF reframes the streams in the garbage can model as problem streams, policy streams (solutions), political streams (participants such as interest groups and government), and policy windows (choices). Furthermore, the MSF underscores the significance of ambiguity, the necessity of interpreting an issue as a problem, and the role of timing. However, it also highlights the focusing events that initiate the process and the policy entrepreneurs and participants who advocate a specific solution. This is in contrast to the garbage can model, in which the actual outcome is more random and the participants are regarded as less entrepreneurial.

In this paper, we will present the argument that several of the aforementioned elements were in play with regard to the skateboard ban. The ambiguity of the problem at hand—namely, whether it is a sports equipment or toy issue—the solution, which took the form of the new Product Control Act, and the timing, when the pressure to make a decision about the “new” skateboarding trend coincided with the passing of a new law, all contributed to the complexity of this issue. Additionally, the fluid participation, with multiple individuals from different departments and organizations coming and going, and the incomplete preferences of the participants, who chose what was seen as the appropriate solution, further complicated matters. Furthermore, the unclear technology, with its diffuse legal boundaries, further exacerbated the situation. These characteristics collectively provide a context for understanding the skateboard ban in Norway. Moreover, we will contend that the disparate streams of problems, solutions, and participants were intertwined, ultimately culminating in the observed outcome.

### Methods and data

The methodology employed in this study is a qualitative analysis of the content of various sources pertaining to the prohibition of skateboarding. As it is near 50 years since the ban was introduced, the material used in this study is from documentary sources, newspaper articles, laws and regulations regarding the ban, historical accounts and, in addition, other scientific work on skateboarding. The following sources have been utilized in this study:

• A significant source is a television documentary from 2006, entitled “Brettkontroll” (Board Control), directed by Emil Trier (22). In the documentary, both bureaucrats and skateboarders are interviewed about the skateboard ban.
• Another important source is a podcast on the history of the ban against skateboarding, “Historien om forbudstida i norsk skating”, [The history of the Norwegian skateboard ban] from 2023, produced by the Norwegian Broadcasting Company, NRK (23).
• To gain a comprehensive understanding of the historical context, a search was conducted in Atekst Retriver, an electronic archive of Norwegian newspaper articles dating from 1960 to the present. The search terms employed were “rullebrett (the Norwegian term for skateboard) og forbud (ban)” and “skateboard og forbud”. A total of 326 results were retrieved, of which 12 were deemed relevant. In this context, the term “relevant” is used to indicate that the article in question directly addresses or justifies the skateboard ban.
• The legislation and regulatory framework pertaining to the prohibition of skateboards: The regulations establishing a ban on the use of skateboards were enacted on September 7, 1978, by the Ministry of Environment in conjunction with the Act of June 11, 1976, concerning the regulation of products (Produktkontrolloven). Furthermore, several reports to the Parliament (white papers) that mention the regulation of skateboards have been reviewed.
• Additionally, an examination of the history of the Statens forurensingstilsyn (SFT) (Norwegian Pollution Control Agency), the governmental agency responsible for enforcing the skateboard ban, is a crucial element in this analysis (24).

As this is a document study, it is necessary to rely on secondary sources in which key players^1^ are interviewed, including documentaries and newspaper articles, to obtain the relevant information. Thus, it is necessary to refer to the selection of quotes made by the directors and journalists. To ensure that the narrative presented is not merely a reproduction of the directors' or journalists' perspective, it has been essential to cross-check the quotes from the different sources against the political and legal documents pertaining to the case and historical accounts.^2^ Moreover, the objective of the data collection is not to create a comprehensive representation of each argument, but rather to ensure that each significant argument is adequately addressed. We identify three primary arguments: firstly, that the prohibition was a moral panic response to a novel and unfamiliar alternative culture; secondly, that it was a reaction to the perceived high risk of injury; and thirdly, that it was a consequence of political processes and categorization. It is important to note that these perspectives are not mutually exclusive, but rather complementary. That is to say, when public officers claim that the regulation of skateboarding is about risk management, this statement is, in and of itself, accurate. However, when the result was a total ban, it is evident that other mechanisms also came into play.

It will be argued that the prohibition of skateboarding may be regarded as an exemplar or “typical case” (23) of the political climate at that time, and thus also refers to the prevailing opinion on what should be seen as proper sport. In this way, the paper offers insight into the sociology of sport, particularly with regard to the criteria for inclusion or exclusion within the field of sport. Furthermore, the prohibition on skateboarding can be regarded as an illustrative example of a complex process, in which the outcome may not necessarily be a result of rational political procedures. Consequently, it offers insights into the extensive body of literature on policy decision-making.

## Risk management or moral panic?

As previously stated, there are two primary explanations for the implementation of the ban. From the perspective of risk management, as espoused by the relevant governmental authorities, and from the perspective of the skateboarders themselves, who have offered a narrative about “moral panic”.

In the United States, the sale of skateboards exhibited a consistent upward trajectory throughout the 1970s. However, skateboarding was not a prevalent activity in Norway during the 1960s and 1970s. Nevertheless, skateboarding garnered attention in Norwegian media, predominantly in a negative light. In the inaugural article on skateboarding in Norway from 1965, published in the Norwegian newspaper Dagbladet, the US correspondent described skateboarding as a “hangover sport”, noting that it was common practice to “skateboard after a party and often in the early morning hours in slalom tracks made up of empty beer cans” (24). In the mid-1970s, media outlets began to inquire whether skateboarding was a healthy activity. A number of articles made reference to statistics from the United States indicating that 75,000 children had sustained injuries while engaged in skateboarding activities. The Norwegian media reported that in seven cases, the donor in kidney transplantation was a child who had died in a skateboarding accident. Furthermore, US health authorities predicted 375,000 injuries, 50,000 hospitalizations, and 50 yearly deaths from skateboarding (24). The small number of skaters in Norway at the time appeared to be largely unconcerned about these developments. Nevertheless, the bureaucrats at the Directorate of Public Roads were cognizant of the statistical data pertaining to skateboarding and noted that in the United States, skateboards had ascended to the second position on a list of “dangerous products”.

On December 8, 1976, the Directorate of Public Roads transmitted a missive to the Ministry of Transport and Communications, inquiring as to whether the recently proposed “product control act” might be employed to regulate skateboarding. The case was subsequently referred to the Consumer Council, which then forwarded it to the Product Control Board. The latter was constituted of representatives from the aforementioned Council, the Norwegian Confederation of Trade Unions, the public sector, and the Norwegian Society for the Conservation of Nature. The Product Control Board proposed that the importation, sale, promotion, and use of skateboards should be prohibited. As the Product Control Act was under the jurisdiction of the Ministry of the Environment, it was this ministry that passed the act, with the Norwegian Pollution Control Authority^3^ serving as the executive authority.

The prohibition of skateboarding was formally enacted on 15 March 1978. This was a provisional prohibition, pending the enactment of a definitive regulation concerning the permissibility and terms of skateboarding (27). However, on 7 September 1978, the provisional ban was superseded by a permanent one (Regulation on the ban of use of skateboard^4^). The ban was subsequently enforced. Individuals engaged in skateboarding during this period frequently reported being pursued by law enforcement, issued fines, and having their boards confiscated. It is challenging to ascertain the frequency of these occurrences, as they constitute a pivotal aspect of the “skateboard narrative”. Nevertheless, it is evident that such occurrences did indeed take place on occasion. In 1979, the white paper on product control work noted that there had been a limited number of infringements resulting in fines, along with a somewhat higher number of instances where skateboards were seized (28).

The rationale behind the prohibition was to avert the occurrence of accidents that are frequently associated with skateboarding. In an advertisement published in Norwegian newspapers in the fall of 1978, the Product Control Board and Pollution Control Authorities informed the public about the ban on skateboarding. The advertisement stated, “Experiences from other countries indicate that uncontrolled growth in the sale and use of skateboards may have adverse consequences and may result in numerous serious accidents, particularly among children”. In an interview, Kjersti Graver of the Consumer Council states, “When this legislation was enacted, it was based on the premise that products should not cause harm to individuals or the environment” [quoted in (22)]. In other words, the government perceived the skateboard as a potential hazard to the health and safety of young people. International statistics and anecdotal personal experiences were among the factors that contributed to this perception. In an interview in the newspaper VG from 1980, Police Inspector Jostein Bovik of the Stavanger Police force stated, “I have personally tested one of these boards in the hallway at the station. It is beyond dispute that this is dangerous equipment” (29).

Outwards in the 1980s, the prohibition on skateboarding was gradually relaxed. Skateboard clubs were permitted to import and sell skateboards, and skateboarding was permitted at facilities designated for that purpose. The clubs were provided with public financial assistance, and skateboarders conducted demonstrations at locations such as art institutions. Skateboarding began to be accepted by the general public. It can be argued that both the increased familiarity with skateboarding and the change in political climate in the 1980s, which saw a shift towards a more liberal political orientation, played an important role in this development. In 1989, despite opposition from the Ministry of Children and Families, the prohibition on skateboarding was lifted.

In the years following the lifting of the ban on skateboarding, the practice has frequently been the subject of ridicule and depicted as an example of unwarranted governmental intrusion into the lives of the general public. However, from the perspective of the legislators at the time, this was primarily a matter of regulating risks. The rationale was that skateboards were dangerous products that are unnecessary and that it is therefore unwise to provide children with unsafe toys. Thus the prohibition of skateboarding can be interpreted as an example of risk management in a comprehensive welfare state (9).

The small number of skateboarders in Norway during the mid to late 1970s undoubtedly perceived the ban in a manner distinct from that of the bureaucrats. From their perspective, the prohibition was perceived as an unwarranted encroachment upon an irreverent cultural practice. In the documentary Brettkontroll, Torgny Amdam, a skateboarder from the era of prohibition, offers the following insight:

The prevailing sports ideal in Norway at the time was comprised of activities such as skiing, football, and hockey, which were viewed as part of the national culture. However, an unexpected phenomenon emerged: the skateboard. This seemingly innocuous object was suddenly perceived as a potential source of injury, particularly in relation to spinal cord and head trauma. What recourse does a bureaucrat have in such a situation? The only recourse is to invoke the act and hope for the best (22).

In alignment with this line of thinking, Øystein Greni, a prominent Norwegian musician and European skateboarding champion in1991, discussed the skateboard ban on a NRK radio program and asserted, “Norwegians are severely misguided. A fear of change and new things” (23). Both quotations underscore the purported outsider or deviant characteristics associated with skateboarding. This understanding of skateboarding is also reflected in the academic literature on the subject. Beal (30) posits that skateboarding represents a form of resistance to capitalist social relations and competition. Gazares (31) states that skateboarding challenges the neoliberal values inherent to the sport of skateboarding itself and provides “spaces of hope”. As mentioned earlier, the literature on legal aspects of skateboarding often relies on understandings that underpin oppositional and irreverent elements that is unwanted in urban areas (1, 5–7). Dickinson et al. (2), for example, argue that skateboarding is illegal in certain urban areas because it challenges the neoliberal ideal of urban development. However, as Lombard (32) argues, it is not possible to claim that skateboarding is inherently resistive. Moreover, Donnelly (33) posits that the romanticization of skateboarding by sociologists has overshadowed its more nuanced oppositional elements. Similarly, Gilchrist and Osborn (34), argues that legal approaches to lifesport is often interpreted as unwarranted and negative disciplination of transgressive cultures. They propose a more nuanced understanding of regulation of lifestyle sports. Nevertheless, as evidenced by the quotations from Norwegian skateboarders above, they perceive themselves as embodying an alternative culture and view the prohibition in this context. In other words, they perceive the prohibition as a manifestation of a moral panic, indicating that the government was unable to cope with the emergence of this new, irreverent culture. As previously stated, our argument is that both the government's assertion that the ban was based on rational risk management and the skateboarders' view that the ban was a form of moral panic fail to sufficiently explain the political mechanism behind the total ban.

## The ban on skateboard as a multiples stream process

### Fluid participation and diffuse responsibilities

In the 1973–74 period, the Department of the Environment commenced its preparations for the new law on product control. The impetus for the legislation was the mounting apprehension about the pervasive contamination by synthetic substances, which was particularly prevalent in Norway and numerous other nations during the 1960s and 1970s. The trade union (LO) advanced the position that the scope of the legislation should extend beyond products that could potentially harm the natural environment and ecosystems to encompass products that could also pose a risk to workers. In the course of preparing the legislation, the issue of product safety was also addressed. The Consumer Council was adamant that this should be incorporated into the legislation. Therefore, despite the initial intention of the act to address environmental concerns exclusively, it ultimately encompassed the regulation of products in general.

The act was implemented on June 11, 1976, and came into effect on September 1, 1977. The legislation applies to all products that have the potential to cause harm to human health or the environment. The legislation imposes a duty of care on all parties involved in the production, importation, processing, distribution, utilization, or any other handling of a product. The legislation empowers the relevant authorities to establish regulatory frameworks, including approval and prohibition schemes, with the objective of preventing damage to health or the environment and ensuring the availability of pertinent information to facilitate the enforcement of the act. Furthermore, regulations were established regarding the supervision of the act's implementation and the establishment of a Product Control Council (22).

Since the act from the outset was meant to handle environmental issues, it was the ministry of the environment that was responsible for handling it. Further government authorities that where involved was The Product Control Council and the Norwegian Pollution Control Authority: “The Product Control Council is subordinate to the Ministry of the Environment and assists the ministry in the implementation of the act. The Norwegian Pollution Control Authority is both an independent product control authority and the secretariat for the council” (27). The Product Control council consisted of 12 members from a range of governmental agencies and NGO's. Their main task was to put forwards proposals for new regulations and to “draw the main lines for the work of the Norwegian Pollution Control Authority” (27).

That meant the Norwegian Pollution Control Authority (SFT) got the responsibility to execute the skateboard act. However, the working force at SFT consisted of people with technical- and natural sciences backgrounds, meant to deal with environmental matters. In report on the history of SFT we can read that “A significant portion of the work—especially the work on product safety—affected individual consumers to a much greater extent than most of what the Norwegian Pollution Control Authority (SFT) otherwise dealt with. (…) For SFT, the responsibility for product control involved new ways of working”. (22).

As illustrated by the quotations below, the SFT were somewhat taken aback when the prohibition on skateboarding was designated as the inaugural case under the new Product Control Act. Rolf Bjørnstad, the director of SFT, states that SFT attempted to suggest alternative solutions to a complete prohibition:

This was probably not the issue we would have prioritized ourselves. However, SFT had to address the cases raised by the Product Control Council. (…) SFT tried to suggest other solutions besides a total ban. This required the involvement of the road authorities or the police. However, they were reluctant to engage, and a large majority in the Product Control Council supported a total ban. At this early stage in our operations, we at SFT found it difficult to go against the Council. This was the case not only in this matter but in others as well. “Therefore, we forwarded the Council's majority decision to the Ministry of the Environment without comment. A total ban on the use of skateboards was then implemented”. [Bjørnstad quoted in (22)].

Later in in the same passage, Bjørnstad also pointed out that it was tricky for a young government agency like STF to find a balance between individual liberty and society's responsibilities: “It is not given that STF had balanced correctly. There were a lot of people inside and outside the environmental protection administration who were wondering: was this really what they had in mind when the law was passed?” (same place). Subsequently, however, he asserted that a total prohibition “were the easiest way to deal” with the regulation of skateboarding and “would pose the least burden” to the government [Bjørnstad in (22)].

The considerable number of government agencies involved in this matter can be regarded as an illustration of diffuse responsibilities. The primary agency, the SFT, lacked the personnel with the requisite expertise to address the full range of products in question. Consequently, the SFT was compelled to defer to the Consumer Council's decisions. The fluidity of responsibilities in this case is exemplified by the following excerpt from a Norwegian newspaper (Aftenposten) from 1984. A journalist made a telephone call to the police and inquired as follows (35):

Why is it not allowed to ride a skateboard?
– Can you explain yourself in more detail?
– Rullebrett (the Norwegian word for skateboard)
– One moment … No, we have nothing to do with that. You must call the Directorate of Roads.
And the Directorate of Roads responds:
– No skateboards…, it comes under the Norwegian Pollution Control Authority
[The journalist then calls the Norwegian Pollution Control Authority]
– Is it your agency that deals with skateboarding?
– That's right.

In addition to the complex network of agencies with ambiguous responsibilities, another factor contributing to the prohibition of skateboards was the prevailing emphasis on safeguarding children from potentially hazardous products. In a government white paper from 1977/78, it was emphasized that the protection of children should be a primary concern (27). In its rationale for the skateboard ban, the Ministry of the Environment cites Report to the Parliament nr 86, which emphasizes the prioritization of children as a user group. “The ban that is now being implemented is therefore clearly grounded in this white paper, according to the ministry” (36).

Rather than viewing this as an instance of moral panic or rational risk management, it can be situated within the framework of what organizational theorists refer to as the logic of appropriateness. In alignment with the perspectives articulated by March and Olsen (17), decision-makers are inclined to prioritize the expectations of their social collective and the perceived appropriateness of a given situation over the pursuit of rational arguments. The Pollution Control Council did not initially *prioritize* the prohibition of skateboarding. However, it was ultimately seen as the *easiest* way to regulating skateboarding in line with the expectations of the wider social collective.

To summarize, skateboarding constituted the inaugural case to be adjudicated under Norway's recently enacted Product Control Act. This development proved a source of surprise for the State Pollution Control Authority (SFT), which, due to the preponderance of its expertise lying in the domain of environmental matters, was not well-prepared to assume the responsibilities inherent to product control. The SFT initially sought alternatives to a total ban but was ultimately compelled by the Product Control Council's majority decision, the reluctance of other actors to engage, and the emphasis on protecting children, as outlined in the White Paper St. Meld. 86., to provide support for a complete prohibition. This decision-making process demonstrated the complex and diffuse responsibilities across a network of organizations, including the SFT, the Ministry of the Environment, the Product Control Council, and others. Moreover, the prohibition was perceived as the *easiest*, though maybe not the most rational, course of action for the authorities.

### Ambiguous categorization

An additional rationale for the prohibition is the ambiguous categorization of skateboards and skateboarding. In the Parliamentary Report on the work with product control in 1977 (27), the term “skateboard sport” is employed in a summary of the process related to the enactment of the ban on skateboarding. Nevertheless, those involved in the preparatory process leading up to the ban emphasized that skateboarding was “play” for children, and thus the skateboard was treated as a toy.

In an interview with the Norwegian newspaper Aftenposten (37), Department Engineer Harald Hæreid, representing the Department of Production Control, stated: “The rationale behind the ban is to prevent the accidents that frequently occur when *playing* with skateboards. Based on experiences from abroad, it can be concluded that *playing* with skateboards has resulted in numerous serious accidents” (italicized by us). Furthermore, as previously stated, the Ministry of the Environment provided an explanation of the ban that the work to protect *children* as a user group should be prioritized, as emphasized in Parliamentary Report 86 (36).

It appears evident that throughout the preparatory work, the perception of skateboarding as play for children prevailed, rather than as a sport. Furthermore, when the skateboard is preceived as toy, it imbued the act of risk management with a certain degree of significance. It is, after all, a parent's natural inclination to ensure that the toys they provide for their children are safe and free from potential hazards. A quote from a police inspector ten years after the ban was passed serves to exemplify this point. “Young skateboarders must be aware that the police have the authority to confiscate their dangerous *toy*” (38). The political administration of the situation pertained to the regulation of a product, as opposed to a sport or cultural practice. If skateboarding had been perceived as a sport, it would have been considerably more challenging to enact the ban. This interpretation is reinforced by the repeal of the regulation banning skateboards in 1989. At this point, skateboarding was reclassified as a sport, aligning it with other sport activities. Accordingly, the “Norwegian Pollution Control Agency (SFT) then placed significant emphasis on “promoting” skateboard use as a challenging and sophisticated sport” (39).

What factors led to the initial perception of skateboarding as not a sport with risk of injuries but play for children? One possible explanation for this discrepancy is the traditional sport culture in Norway. The fundamental tenets of Norwegian sporting practice have historically placed an emphasis on endurance and strength, which are inextricably linked with cross-country skiing. The cultural legacy of Norwegian national sport, “the sport of all sports”, has shaped Norwegians' conceptualization of what constitutes proper sport (40). This ascetic perspective on the nature of sportsmanship has been in opposition to the more playful and hedonistic view of sport (41). From this, it can be inferred that from the perspective of bureaucracy, skateboarding was perceived as a form of play rather than a sport.

### A solution searching for a problem

Timing is of paramount importance in a multiple-stream approach (20). The history of the Product Control Act began several years earlier, initially focusing on chemical pollution and subsequently expanding to encompass a range of harmful products and substances, including those used in workplaces and for leisure activities. SFT, which coincidentally became the responsible agency for the act, had traditionally been concerned with pollution of nature and the environment. As a result, the product of the skateboard fell outside the remit of the agency, as we saw above. Concurrently, the skateboard phenomenon became increasingly conspicuous, giving rise to a number of concerns. The Director of Public Road then inquired of the Ministry whether a prohibition on skateboards could be enacted under the new product control act that was under preparation. Consequently, the regulation of skateboards became the inaugural case under the new act for STF. It served as a benchmark for an act with perceived ambiguous boundaries. In this context, it can be argued that the *solution*, namely the Product Control Act, sought to identify and address a *problem*: the emerging trend of skateboarding that coincided with the passing of the act (16).

In conclusion, this section has proposed that the political process surrounding the Product Control Act is a vital prerequisite for the prohibition of skateboarding in Norway. The process was characterized by fluid participation and nebulous responsibilities, which ultimately gave rise to uncertainty regarding the appropriate course of action. Initially, the case was treated as a temporal prohibition, pending the development of regulations. However, it was subsequently deemed to be a prolongation of a permanent ban. This suggests that the solution selected may have been the simplest one, while appearing to be appropriate, may not have been the most optimal. The ambiguity surrounding the nature of the problem—whether childrens play or an expanding sport culture, whether a toy or a sporting equipment—led to the prioritization of risk management as a means of addressing the former. The coincidence between this solution—the Product Control Act—and the problem was crucial. The convergence of a series of political streams, the rising popularity of skateboarding, the enactment of legislation initially designed to address a different issue, and the involvement of numerous government actors with limited or no expertise on the matter collectively led to the prohibition of skateboarding.

## Conclusion

In this paper, we have put forth the argument that the prohibition of skateboarding was less an instance in which a patronage state sought to regulate and oppose an oppositional culture. The motivation to regulate skateboarding was primarily risk management. Nevertheless, the ultimate result—a complete prohibition—was largely shaped by the political process preceding the Product Control Act and the subsequent regulation of skateboarding. It appears that skateboarding has become enmeshed in a fortuitous but unintentional web of circumstances, a complex system devoid of any discernible guiding force. The primary elements of this complex web can be summarized as follows:

(1) This was the inaugural case to be addressed by an entirely novel legislative instrument.
(2) The act, initially focused on the natural environment, subsequently expanded to encompass product control. However, it was not designed to regulate the urban environment. In other countries, legal instruments such as property law and urban regulations were employed to regulate skateboarding.
(3) The agency responsible, the Norwegian Pollution Control Authority, had a staff with experience in addressing environmental concerns.
(4) The issue involved a considerable number of governmental agencies, some of which were reluctant to assume responsibility.
(5) A new white paper exerted pressure on the government to prioritize the safety of children.
(6) Skateboards were categorized as toys, not sports equipment.

As previously stated, the prohibition has been the subject of ridicule both at the time of its implementation and in the subsequent period. In this context, it can be argued that Norway's approach to regulating electric scooters the last decades is relatively liberal compared to other European countries. By allowing the use of these vehicles, the government avoids being seen as hypocritical and avoids being subjected to the same level of criticism it faced over the skateboard ban.

## Data Availability

The original contributions presented in the study are included in the article/Supplementary Material, further inquiries can be directed to the corresponding author.

## Footnotes

^1^Who are now, 47 years later, either retired, in care or dead.

^2^To our knowledge there is no digital archive of the correspondence, internal notes etc. from that period; only Report to the Storting (withe paper), Proposition to the Storting, and Norwegian Official Report are digitalized.

^3^Now a part of the Norwegian Environment Agency.

^4^Determined by the Ministry of Environment, in agreement with the Product Control Act of June 11, nr. 79.
